# Recent Development of Fibrous Hydrogels: Properties, Applications and Perspectives

**DOI:** 10.1002/advs.202408657

**Published:** 2024-11-12

**Authors:** Wen Luo, Liujiao Ren, Bin Hu, Huali Zhang, Zhe Yang, Lin Jin, Di Zhang

**Affiliations:** ^1^ International Joint Research Laboratory for Biomedical Nanomaterials of Henan Henan Key Laboratory of Rare Earth Functional Materials Zhoukou Normal University Zhoukou 466001 P. R. China; ^2^ The Key Laboratory of Biomedical Information Engineering of Ministry of Education School of Life Science and Technology Xi'an Jiaotong University Xi'an 710049 P. R. China; ^3^ Research Institute of Xi'an Jiaotong University Hangzhou 311200 P. R. China; ^4^ Department of General Surgery (Colorectal Surgery) Guangdong Provincial Key Laboratory of Colorectal and Pelvic Floor Diseases Guangdong Institute of Gastroenterology Biomedical Innovation Center, The Sixth Affiliated Hospital Sun Yat‐sen University Guangzhou 510655 P. R. China

**Keywords:** conductivity, ECM‐like, fibrous hydrogels, mechanical, stimulus

## Abstract

Fibrous hydrogels (FGs), characterized by a 3D network structure made from prefabricated fibers, fibrils and polymeric materials, have emerged as significant materials in numerous fields. However, the challenge of balancing mechanical properties and functions hinders their further development. This article reviews the main advantages of FGs, including enhanced mechanical properties, high conductivity, high antimicrobial and anti‐inflammatory properties, stimulus responsiveness, and an extracellular matrix (ECM)‐like structure. It also discusses the influence of assembly methods, such as fiber cross‐linking, interfacial treatments of fibers with hydrogel matrices, and supramolecular assembly, on the diverse functionalities of FGs. Furthermore, the mechanisms for improving the performance of the above five aspects are discussed, such as creating ion carrier channels for conductivity, in situ gelation of drugs to enhance antibacterial and anti‐inflammatory properties, and entanglement and hydrophobic interactions between fibers, resulting in ECM‐like structured FGs. In addition, this review addresses the application of FGs in sensors, dressings, and tissue scaffolds based on the synergistic effects of optimizing the performance. Finally, challenges and future applications of FGs are discussed, providing a theoretical foundation and new insights for the design and application of cutting‐edge FGs.

## Graphical abstract

This review systematically analyzes the enhance properties of Fibrous hydrogels, including the enhanced mechanical properties, high conductivity, high antimicrobial and anti‐inflammatory, stimulus responsiveness, and ECM‐like structures. Additionally, a detailed analysis explores how the synergistic effects among optimized properties influence the applications of FGs as sensors, dressings, tissue scaffolds and others.

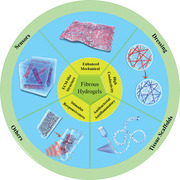

## 1 Introduction

Fibrous hydrogels (FGs) are elastic materials formed by prefabricated fibers, fibrils, and polymers, which create a 3D network structure through physical entanglement or chemical cross‐linking. They exhibit a high compatibility with biological tissues and excellent multifunctionality.^[^ ^1^, ^2^, ^3^, ^4^ ^]^ The internal fiber structure enhances flexibility, elastic stretch, and anti‐swelling,^[^ ^5^, ^6^, ^7^ ^]^ as well as promotes cell adhesion, proliferation, differentiation, migration, and signal transduction.^[^ ^8^, ^9^, ^10^ ^]^ Consequently, they have emerged as ideal next‐generation biomimetic materials. Over the past decade, FGs have undergone rapid advancements.^[^ ^11^, ^12^ ^]^ The design approaches of FGs have evolved from simple assembly to multifunctional. The application fields have extended from in vitro cell culture environments to various domains such as the spinal cord, tendons, bones, and heart. By amalgamating the advantages of fibers and hydrogels, FGs have emerged as prominent materials in multiple fields.^[^ ^13^, ^14^ ^]^

By altering the composition and structure of fibers or hydrogels^[^ ^15^, ^16^, ^17^ ^]^ and employing unique fabrication techniques such as spinning, 3D printing, and microfluidic spinning.^[^ ^6^, ^18^, ^19^, ^20^ ^]^ FGs exhibit advantages such as high mechanical performance, superior conductivity, robust antibacterial and anti‐inflammatory properties, intelligent responsiveness, and ECM‐like structures.^[^ ^21^, ^22^, ^23^ ^]^ Currently, FGs with high mechanical performance exhibit compressive strengths of up to 556.6 MPa, elongation of rates up to 44 200%, and an ultra‐thin thickness of around 3.4 µm.^[^ ^24^ ^]^ The high conductivity of FGs mitigate the trade‐off between mechanical performance and resistance to failure, with conductivity reaching up to 247 S cm^−1^.^[^ ^25^, ^52^ ^]^ FGs with robust antibacterial and anti‐inflammatory properties have significantly improved the precision of on‐demand drug release and data models in large mammalian studies.^[^ ^26^ ^]^ Intelligent responsive FGs can mimic the stimulus‐response mechanisms of biological organisms, thereby enabling the design of materials that sense and respond to the environment.^[^ ^27^ ^]^ FGs with ECM‐like structures can simulate and reconstruct the architecture and functionality of the ECM,^[^ ^28^, ^29^ ^]^ effectively transmitting biophysical and biochemical properties to facilitate the study of cell‐matrix interactions.^[^ ^30^ ^]^ Owing to these advantages, FGs have demonstrated substantial potential for applications in sensors, tissue scaffolds, and wound dressings.

In recent years, with the increasing research interest, several reviews have been published on the preparation and application of FGs. Examples include the preparation of conductive hydrogel fibers and applications in flexible electronics, the design strategy of FG with layered biomimetics, and the application of electrospun fiber hydrogels in tissue engineering, such as blood vessels, nerves, and organ tissues.^[^ ^31^, ^32^, ^33^ ^]^ Summarizing and analyzing the latest performance improvement strategies and applications of FGs will help researchers understand this field more comprehensively. In this review, we focus on the performance regulation strategies of FGs in five aspects mechanical properties, electrical conductivity, antibacterial and anti‐inflammatory properties, intelligent response, and ECM structure. We also summarize different application fields such as sensors, wound dressings, tissue scaffolds, and water purification.

**Figure 1:**
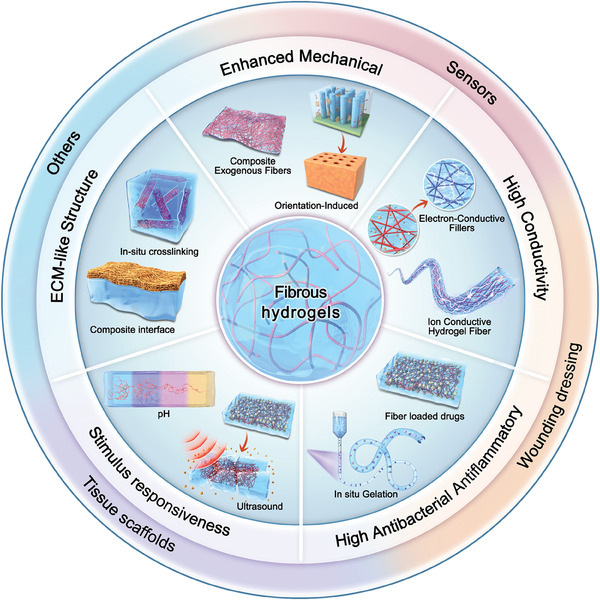
Schematic illustration of regulation properties and applications of fibrous hydrogels.

In this review, we explored three strategies to improve the mechanical properties of FGs: covalent cross‐linking, enhancing the density of the fiber network, modifying the arrangement of fibers, and in situ induction of stratified structures. Subsequently, we discuss three approaches to improve the conductivity of FGs: assembling conductive fillers into a 3D network, crating ion transport channels, and constructing hydrogel fibers. In addition, we elucidated approaches to enhance the antibacterial and anti‐inflammatory properties of FGs, including drugs embedded in fibers and in situ gelation of drugs with exogenous fibers. Furthermore, the intelligent stimulus‐responsive characteristics of the FGs were analyzed from single to multipath response perspectives. The construction of ECM‐like structures in FGs can be realized by four methods: cross‐linking of prefabricated fibers with polymers, interfacial recombination of fibers and hydrogels, molecular modification and assembly, and direct cross‐linking of peptide fibers with polymers. Based on the synergistic effects of the five optimized properties of FGs, we further summarized the breakthroughs of FGs in various applications, including sensors, dressings, tissue scaffolds, and water purification (**Figure** **1**). Finally, there is an overview and outlook on the current challenges and future development trends for multifunctional FGs. Thus, this review provides a fundamental understanding of the principles necessary for designing FGs with optimal performance and offers fresh insights into the prospective applications of FGs.

## 2 Performance Improvement Strategies

FGs mainly consist of two components: fibers and hydrogels. In this study, the fibers were obtained from two main sources. The first is preformed fibers or exogenous fibers obtained through methods such as spinning, stretching, and 3D printing. The other is the in situ assembly of fibrils such as peptides, protein molecules, and cellulose. The hydrogel was formed by cross‐linking polymers. By altering the composition and structure of the fibers, as well as the polymeric materials and methods used in hydrogel synthesis, researchers can modulate and customize advanced FGs. In this section, we discuss strategies for regulating the performance of FGs from five perspectives: enhanced mechanical properties, high conductivity, antibacterial and anti‐inflammatory properties, ECM‐like structure regulation, and stimulus responsiveness.

### 2.1 Enhanced Mechanical Properties

**Figure 2:**
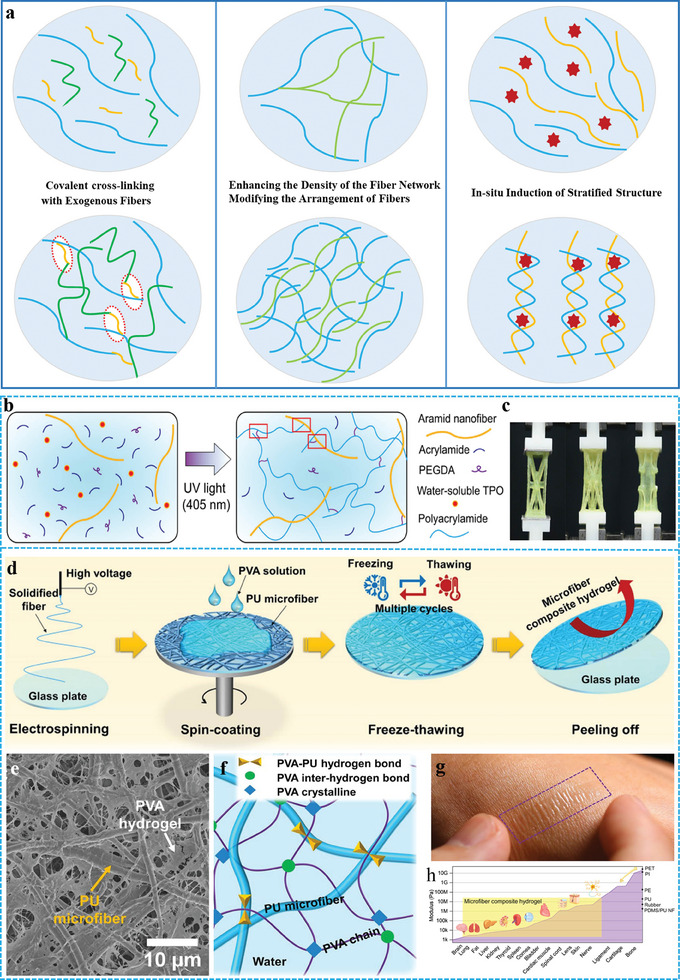
a) Schematic of three strategies to enhance mechanical properties. b) Schematic about the fabrication process of the FGs. c) The photo of the hydrogel in the initial and stretched states. Reproduced with permission.^[^ ^56^ ^]^ Copyright 2023, Elsevier. d) Preparation process and SEM image e) of the hydrogel film. f) Illustration of the PU fibers embedded structure and bonding mechanisms. g) The photo of the FGs film adhere to skin. (h) Modulus matching range of the FGs. Reproduced with permission.^[^ ^24^ ^]^ Copyright 2023, Springer Nature.

Traditional hydrogel materials are typically based on a single water‐soluble polymer chain cross‐linked network. The abundance of solvents leads to a reduction in intermolecular entanglement, resulting in weak mechanical properties and low swelling resistance, limiting them to load−bearing materials.^[^ ^34^, ^35^, ^36^, ^37^, ^38^, ^39^ ^]^ Inspired by the nanofiber network structure of load‐bearing soft tissues in organisms, nanofiber materials that resemble biomacromolecule bundles can be added to hydrogels to obtain FGs with significantly improved mechanical properties.^[^ ^40^, ^41^, ^42^ ^]^ The current challenge with FGs is that the interfacial interaction between the fibers and hydrogel matrices is weak and needs to be optimized.^[^ ^43^, ^44^, ^45^ ^]^ Furthermore, there is a misalignment between the mechanical properties of the hydrogels and their multifunctionality, demanding a balance between maintaining mechanical integrity and preserving multifunctionality.^[^ ^46^, ^47^ ^]^ To address these two challenges, recent breakthrough strategies can be divided into three main types (**Figure** **2**a): i) covalent cross‐linking, ii) enhancing the density of the fiber network and modifying the arrangement of the fibers, and iii) in situ induction of layered structures. The compressive strength of the FGs obtained by the above methods is up to 556.6 MPa, the tensile ratio was up to 44 200%, and the thickness was approximately 3.4 µm. The mechanical properties of typical FGs are listed in **Table** **1**.

**Table 1:** Representative examples of FGs with the mechanical performance.

Main Compositions of FGs	Tensile Strength	Thickness	Strain	Conductivity	Reference
Polyurethane fibers / polyvinyl alcohol	≈6 MPa	< 5 µm	─	─	[24]
gelatin fibers / polyvinyl alcohol	10.12±0.50 MPa	─	142%	─	[48]
Aramid	556.6 MPa	3.4 µm		─	[5]
Cellulose	226 MPa	─	44 200%	─	[49]
polyvinyl alcohol fibers / H_2_O	12.8±0.7 MPa	─	1719±77%	─	[50]
Polyacrylamide / poly(acrylamide‐co‐acrylic acid)	> 4 MPa	─	> 400%	0.84 mS cm^−1^	[51]
polyacrylic acid	─	─	610%	247 S cm^−1^	[52]
silk fibroin	55 MPa	─	530%	0.45 S m^−1^	[88]
Paraffin / polypyrrole / Silver nanowires	≈0.6 MPa	─	≈500%	0.148 S m^−1^	[159]

#### 2.1.1 Covalent Cross‐Linking

The excellent mechanical properties of biological tissues primarily stem from their inherent high stiffness nanofibers and unique microstructures. Therefore, the introduction of stiff exogenous fibers is a promising strategy to enhance the mechanical properties of hydrogels.^[^ ^53^, ^54^, ^55^ ^]^ Owing to the weak interface between fibers and hydrogels, the strong interaction of covalent cross‐linking between exogenous nanofibers and hydrogels is a key problem in achieving the excellent mechanical properties of FGs. Li et al. have introduced arylamide nanofibers (ANFs) as exogenous nanofibers in hydrogels.^[^ ^56^ ^]^ The photochemical modification initiated covalent cross‐linking and hydrogen bonds between the nanofibers and the hydrogel matrix (Figure 2b), which lead to the ANF‐enhanced hydrogels. Compared with the empty hydrogel, the strength, fracture energy, and fatigue threshold of the ANF‐hydrogel composites increased. (Figure 2c) It can be attributed to the formation of a hybrid network of ANF and hydrogel chains during free‐radical polymerization, whereas the increase in fracture energy is primarily related to the energy dissipation mechanism between long‐chain entanglements and a large number of hydrogen bonds.

#### 2.1.2 Enhancing the Density of the Fiber Network and Modifying the Arrangement of Fibers

The low density of the fiber network facilitated the production of fragile and soft FGs. The arrangement of the fiber network has a more direct effect on the mechanical properties of the fiber hydrogel. Therefore, increasing the fiber network density and adjusting the arrangement of the fiber network can improve the mechanical properties of hydrogels.^[^ ^57^, ^58^, ^59^ ^]^ Zhang et al. developed an ultra‐thin microfiber composite hydrogel film by embedding a polyurethane electrospun fiber network in polyvinyl alcohol (PVA) hydrogel.^[^ ^24^ ^]^ (Figure 2d) Enhancing the density of the fiber network and modifying the arrangement of the fibers provide FGs with outstanding mechanical strength and tear resistance. (Figure 2e–h) The most unique thing is that the additions of glycerol and salt ions ensure that the FGs have high ionic conductivity and outstanding anti‐dehydration behavior. These features further improve the construction of attached flexible bioelectronic devices for monitoring biological signals.

#### 2.1.3 In Situ Induction of Stratified Structure

**Figure 3:**
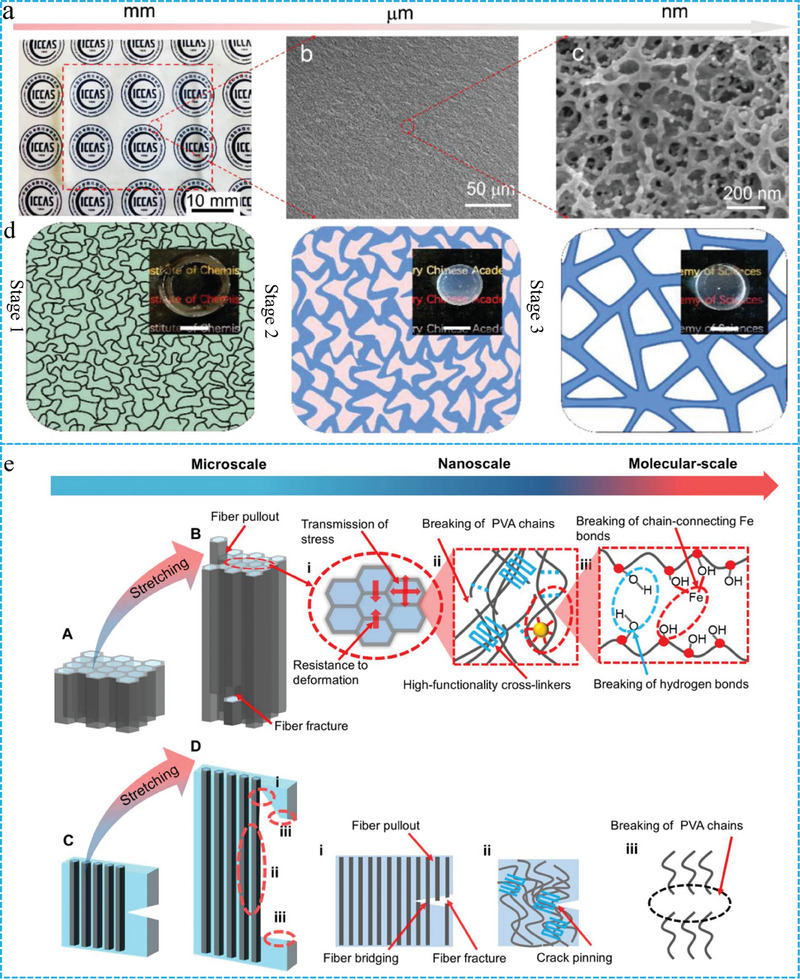
a) images of hydrogel at macroscopic and b,c) microscopic. d) Three stages of solvent‐exchange strategy for the preparation of FGs. Reproduced with permission.^[^ ^66^ ^]^ Copyright 2024, Wiley‐VCH. e) The mechanisms behind performance enhancements the at each length scale. Reproduced with permission.^[^ ^68^ ^]^ Copyright 2024, American Association for the Advancement of Science.

The in situ formation of nanofibers in the hydrogel polymer chains can prevent interfacial incompatibilities and significantly improve the mechanical properties of hydrogels along the orientation direction.^[^ ^60^, ^61^ ^]^ At present, orientation‐induced in situ FGs have certainly improved mechanical properties within a single‐scale anisotropic structure, but a balance between strength and elongation is required.^[^ ^62^, ^63^, ^64^, ^65^ ^]^ Therefore, the addition of a multi‐scale hierarchical structure based on anisotropy is necessary. Qiu et al. constructed FGs in situ using non solvent quenching‐tempering.^[^ ^66^ ^]^ This strategy achieved the controlled aggregation of nanoscale polymer segments by manipulating the solvent without specific orientation. First, non‐ solvent quenching was carried out by freeze casting, and phase separation was driven in situ to increase the interaction of the PVA polymer chains to obtain PVA‐NMP gels. The gels were then solvent‐tempered, and the micro phase of the polymer gradually evolved into nanofibers to form isotropic strong PVA FGs. (Figure 3d) The macro‐ and microscopic morphologies of the FGs are shown in **Figure** **3**a–c. The homogeneous and isotropic network structure makes FGs exhibit significantly enhanced mechanical properties, swelling resistance, and unique self‐reinforcing tensile properties. The introduction of the stratified structure successfully produced FGs with high strength and toughness.^[^ ^67^ ^]^ As research progresses, there is an urgent need to understand the intrinsic mechanisms for improving the mechanical properties of FGs. Establishing the relationship between structure and performance will advance design strategies for FGs and drive their engineering applications. Zhai et al. developed FGs with hierarchical structures ranging from the molecular to micrometer level by immersing oriented frozen PVA in an ethanol solution of ferric chloride.^[^ ^68^ ^]^ The authors utilized theoretical simulations to analyze the mechanisms underlying the performance enhancements at each scale (Figure 3e). This study provides insights into the design of stratified FGs.

The modulus and strength of FGs are improved, but the ductility and toughness are decreased by the covalent cross‐linking method, which can be attributed to the weak interfacial connection between the hydrogels and fibers with different physical and chemical properties. The strong interaction between exogenous nanofibers and hydrogels is a key factor in obtaining excellent mechanical properties of FGs. Enhancing the density of the fiber network and modifying the arrangement of the fibers can avoid interfacial incompatibility, and the mechanical properties of the hydrogel along the alignment direction are significantly improved. However, the mechanical properties and anisotropy of the network structure in the vertical direction are suboptimal. Compared to the previous two methods, the in situ induction of stratified structures addresses the weak interfacial interaction between the fibers and the hydrogel and enables the fabrication of isotropic and multiscale hierarchical FGs. Notably, the toughening mechanism further enhances the design of FGs with superior mechanical properties.

### 2.2 High Conductivity

**Figure 4:**
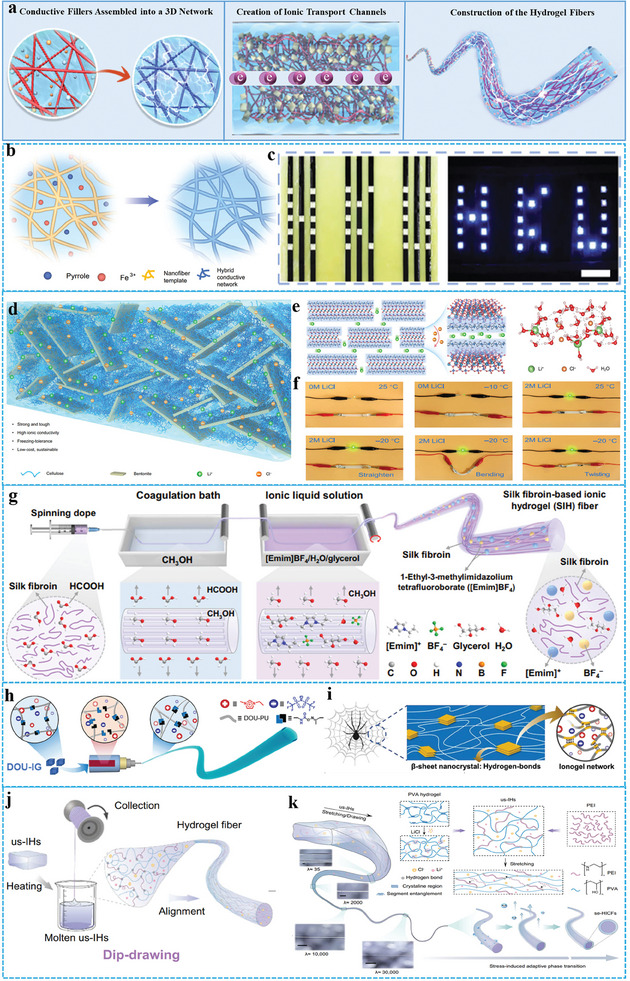
a) Schematic of three strategies to enhance conductivity properties. b) Schematics of the template about nanofibers and the conductivity c) of hydrogel. Reproduced with permission.^[^ ^84^ ^]^ Copyright 2023, Springer Nature. d) Schematic illustrations of the microstructure for the hydrogels. e) Schematic diagram of ions movement. f) The photos about luminance of LEDs by hydrogel at different temperatures. Reproduced with permission.^[^ ^86^ ^]^ Copyright 2022, Springer Nature. g) The preparation process of silk ionotropic hydrogel fibers. Reproduced with permission.^[^ ^88^ ^]^ Copyright 2024, Springer Nature. h) The process of the melt spinning of hydrogel fiber. Reproduced with permission.^[^ ^89^ ^]^ Copyright 2024, American Association for the Advancement of Science. i) Schematic of spider‐silk‐inspired iongel structure. Reproduced with permission.^[^ ^90^ ^]^ Copyright 2024, Wiley‐VCH. j,k) The preparation process of hydrogel fiber. Reproduced with permission.^[^ ^91^ ^]^ Copyright 2024, Springer Nature.

Conductive fiber hydrogels (CFGs) are composed of fibers, polymer networks, water, and electrical and ionic materials. Among them, the polymer and electrical/ionic networks play a crucial role in determining the overall performance.^[^ ^69^, ^70^, ^71^, ^72^ ^]^ Conductivity is a key property used to evaluate the conductivity of CFGs. The conductivity of CFGs is due to the movement of electrons or ions in their network structure.^[^ ^73^, ^74^, ^75^, ^76^ ^]^ Three main directions were used to increase the conductivity of the CFGs. (**Figure** **4**a) i) Conductive fillers assembled into a 3D network: The conductive filler forms a 3D network structure through in situ polymerization under the template of the 3D network, which can improve the electrical conductivity. ii) Creation of ionic transport channels: The channels are crafted in CFGs through π–π stacking, hydrophobic effect, and hydrogen bonds, which can enhance the electrical conductivity. iii) Construction of hydrogel fibers: Hydrogel fibers based on 3D network microstructure can be obtained by continuous spinning to improve ion transport rates.

#### 2.2.1 Conductive Fillers Assembled into a 3D Network

As an important component of CFGs, conductive agents influence conductivity. The addition of suitable conductive agents can build a conductive network and provide a fast channel for electron transmission, thus endowing the conductivity of FGs. Therefore the addition of conductive agents play an important role in the CFGs.^[^ ^77^, ^78^ ^]^ The current incorporation of electronically conductive fillers into hydrogels does not result in composite materials due to the random distribution of the conductive fillers in the hydrogel matrix and the low volume fraction of the conductive phase with the same high conductivity as the fillers, making it difficult to achieve electroosmotic flow.^[^ ^79^, ^80^ ^]^ In addition, increasing the amount of conductive fillers may impair the mechanical properties, water content, and other properties of the hydrogels.^[^ ^81^, ^82^, ^83^ ^]^ Although these properties are critical for functional applications of FGs, there is a trade‐off between high conductivity and strong mechanical properties. Thus, conductive fillers and microstructures play a crucial role in determining the performance of conductive hydrogels. Using the 3D network template effect of nanofibers to assemble conductive fillers, a stable conductive network can be established, endowing the FGs with exceptional mechanical properties and conductivity. Recently, Xu et al. prepared FGs with high conductivity and strong mechanical properties on an aramid nanofiber template by combining polypyrrole and polyvinyl alcohol.^[^ ^84^ ^]^ (Figure 4b,c) This is attributed to the 3D hyperconnected network of nanofibers, which induces the assembly of conductive polymers.

#### 2.2.2 Creation of Ionic Transport Channels

Ionic conductors offer several advantages over electronic conductors, such as flexibility, high stretchability, and transparency.^[^ ^85^ ^]^ Ionic conductive hydrogel fibers exhibit similar conductivity for transmitting bioelectric signals in the human body, with the directed movement of charged ions in the network facilitating conductivity. Currently, there are two primary sources of conductivity in ionic conductive hydrogels: zwitterionic polymers and ions. Free ions are more widely used than zwitterionic polymers owing to their flexibility and distinctive properties. However, a persistent challenge lies in the ease with which ions diffuse into the hydrogel network, weakening the hydrogen bonds between polymer chains and creating a trade‐off between electrical conductivity and mechanical strength. The creation of efficient ion transport channels can address these issues and enhance the conductivity of ionic conductive FGs. Chen et al. used the strong coordination between cellulose molecules and 2D nano‐bentonite to build a reasonable and robust binding network in FGs and obtained a supramolecular cellulose‐bentonite hydrogel (Figure 4d).^[^ ^86^ ^]^ The ionic conductivity of FGs reached 89.9 and 25.8 mS cm^−1^ at 25 and −20 °C, respectively (Figure 4f). This high conductivity is because of the negative charge carried by the cellulose/BT nanocomposites, and the gap between adjacent BT nanosheets serving as a fast‐moving channel for cations. In contrast, LiCl can weaken the hydrogen bonds between water molecules, reducing the freezing point, which ensures that the FGs have high ionic conductivity even at low temperatures. (Figure 4e). This work presents a novel approach for constructing strong and tough all‐natural conductive FGs.

#### 2.2.3 Construction of the Hydrogel Fibers

Compared to traditional bulk or membrane FGs, FGs that combine a 3D microstructure with a 1D macroscopic structure possess continuous electron and ion transport channels, and also shorten the diffusion distances, thereby accelerating the transfer rate of electrons and ions. Recently, ionic conductive hydrogel fibers, that facilitate ion‐mediated signal transmission through the infiltration of ionic liquids, have emerged as a focal point of research.^[^ ^87^ ^]^ Zhang et al. used continuous wet spinning to fabricate silk ionotropic hydrogel fibers that resembled natural silk in their semi‐crystalline and oriented structure (Figure 4g)^[^ ^88^ ^]^ The plasticizing effects of the ionic liquids [Emim]BF_4_, glycerol, and water endowed the fibers with excellent mechanical properties, exhibiting a tensile strength of 55 MPa and an elongation at break of 530%. The incorporation of [Emim]BF_4_ enable the SIH fibers to achieve a stable ionic conductivity of 0.45 S m^−1^. Currently, the development of simple, scalable manufacturing methods and enhancement of stability during use are the two primary challenges for ionic conductive hydrogel fibers. Through continuous melt spinning, molecular covalent cross‐linking, and functional self‐encapsulation, ionic conductive hydrogel fibers with high tensile strength, robust stability, and strong self‐healing capabilities are gradually being developed. You et al. proposed a new molecular strategy based on a dynamic covalent cross‐linking network and used continuous melt spinning to prepare covalently cross‐linked ionic gel fibers based on a dimethyl butanedione dimer carbamate group (Figure 4h).^[^ ^89^ ^]^ This fiber exhibited multiple properties such as high stretchability, transparency, conductivity, solvent resistance, and self‐healing ability. Inspired by the spider silk structure, You et al. proposed a multi‐level hydrogen bonding strategy to produce robust, self‐healing, easy‐to‐process, and recyclable multifunctional ionic gels (Figure 4i).^[^ ^90^ ^]^ The synthetic ionic gel displayed spider like characteristics and hysteresis behavior, indicating excellent energy dissipation performance. Li et al. proposed a stress induced adaptive phase‐change strategy for the continuous preparation of highly stable conductive fibers (Figure 4j).^[^ ^91^ ^]^ This process consists of only two steps: self‐encapsulation based on an adaptive phase change and stress‐induced tensile/dip pull formation (Figure 4k). Ionically conductive hydrogel fibers with stable packaging structures were obtained through an environmentally friendly and efficient strategy.

Conductive fillers assembled into 3D networks have been shown to be effective in balancing the high conductivity and robust mechanical properties of conductive FGs. However, this approach exhibits selectivity toward templates for 3D networks and requires stringent reaction conditions, which impose certain limitations on its practical application. Similarly, the creation of ion transport channels can yield strong and resilient conductive FGs, although they lack broad applicability. The third approach produces transparent, high‐aspect‐ratio, and highly wearable ionic conductive hydrogel fibers, making them promising candidates as soft materials for flexible electronic technologies. However, owing to the poor spinning ability of the hydrogel and its precursor solution, the large‐scale manufacturing of fibers remains a challenge. Moreover, ionic conductive hydrogel fibers tend to dehydrate and deionize during use, so enhancing their stability becomes critically important. For commercial application, the integration of multiple functionalities into devices based on ionic gels, as well as large‐scale production and popularization, remains the focus of ionic conductive hydrogel fibers.

### 2.3 High Antibacterial and Anti‐Inflammatory

**Figure 5:**
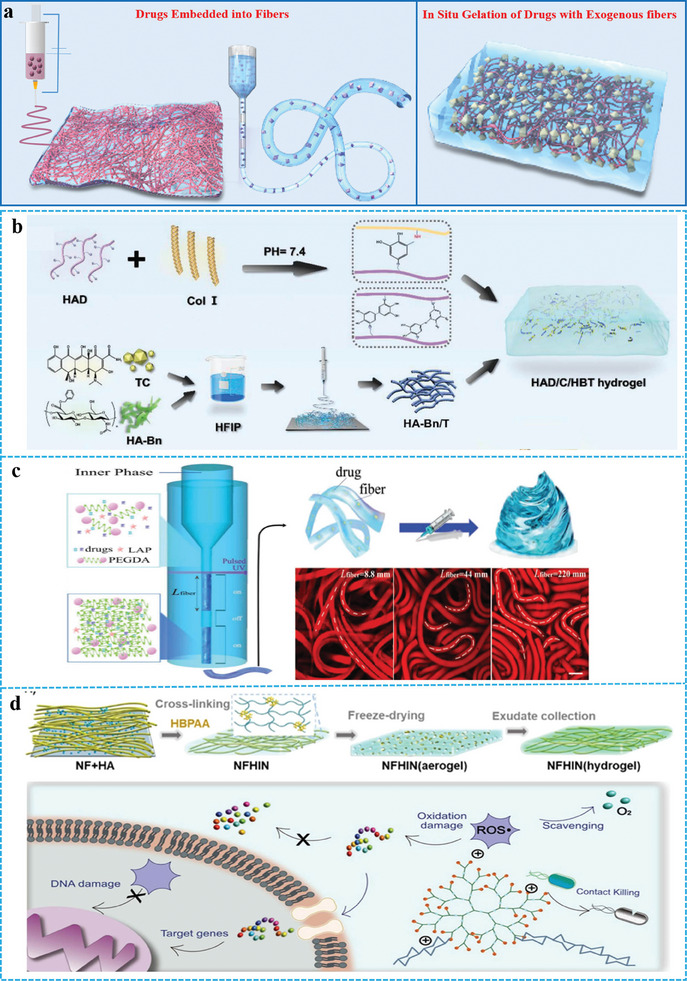
a) Schematic of two strategies for high antibacterial and anti‐inflammatory. b) Schematic illustrations of FGs generation. Reproduced with permission.^[^ ^102^ ^]^ Copyright 2023, Wiley‐VCH. c) Schematic diagrams of the preparation route to FGs. Reproduced with permission.^[^ ^103^ ^]^ Copyright 2023, American Association for the Advancement of Science. d) Schematic diagrams for the synthetic route and mechanism of polycationic hydrogel with exudate. Reproduced with permission.^[^ ^105^ ^]^ Copyright 2023, IOP Publishing.

Photothermal and drug therapy are two main pathways of antibacterial and anti‐inflammatory effects, respectively. The mechanism of photothermal therapy allows the prevention of drug resistance during the treatment of bacterial infections.^[^ ^92^ ^]^ Zhou et al. designed a multifunctional hydrogel dressing with mild photothermal and antioxidant properties for wound healing. The dressing can release Zn^2+^ under irradiation with light in the near infrared range and can achieve an effective antibacterial effect under light and heat.^[^ ^93^ ^]^ However, owing to the lack of specificity towards bacteria, this therapy tends to cause damage to host cells when killing bacteria, and its application is only suitable for the in vitro stage.^[^ ^94^, ^95^ ^]^ Compared to photothermal therapy, the addition of antibacterial drugs to FGs are more commonly used.^[^ ^96^, ^97^ ^]^ Moreover, drugs loaded FGs can stably release drugs on demand and exhibit intelligent and stable antibacterial and anti‐inflammatory properties.^[^ ^98^, ^99^ ^]^ The ways to obtain antibacterial and anti‐inflammatory FGs can be divided into the following two categories (**Figure** **5**a):

#### 2.3.1 Drugs Embedded into Fibers

Owing to their hydrophobicity, most antibacterial and anti‐inflammatory drugs exhibit uneven dispersion in FGs. In addition, the large pore size of the hydrogel leads to the premature release of small molecule drugs, which is not conducive to sustained antibacterial effects. Electrospinning can be used to easily and effectively prepare ECM‐like nanofibers. Drugs are embedded into nanofibers by electrospinning, which can achieve effective drug loading, control the drug release rate, and prevent premature burst release of drugs.^[^ ^100^, ^101^ ^]^ Sun et al. have obtained tetracycline‐loaded esterified hyaluronic acid nanofibers by electrospinning. The fibers were chemically interwoven with dopamine‐modified hyaluronic acid to form a stable FG. This FG retains the functional protein environment and inhibits bacterial infection and regulate wound inflammation. (Figure 5b).^[^ ^102^ ^]^ The current status of FGs with antibacterial and anti‐inflammatory properties is as follows: i) The mechanical properties of FGs have an important impact on the ECM phenotype of cells, but they lack precise customized mechanical properties to meet specific cell regeneration needs. ii) Adjustable drug release rate plays an important role in drug efficacy, and intelligent FGs that can release suitable molecules at different rates are urgently required. Therefore, controllable hydrogel materials can provide customized treatment procedures and drug release curves at rates suitable for different biological applications, which will help improve their antibacterial and anti‐inflammatory properties. Shum et al. used a continuous microfluidic method to blend the polymer phase with a photoinitiator, lithium phenyl‐2,4,6‐trimethylbenzoylphosphinate (LAP), and pharmaceuticals, which subsequently flowed into the inner phase.^[^ ^103^ ^]^ A co‐flow configuration was established using salt as the outer phase. Gelation was facilitated by pulsed UV irradiation, and the fiber length was adjusted via exposure time. FGs are extruded from the needle, and as the length of individual microfibers (L fibers) increases from 8.8 to 220 mm, the quantity of looped and coiled fibers grows, leading to a more intricate topological entanglement of the FGs (Figure 5c). This technique offers a novel method for the precise customization of gels for in situ tissue engineering applications.

#### 2.3.2 In Situ Gelation of Drugs with Exogenous Fibers

For hydrophilic drugs, antimicrobial and anti‐inflammatory FGs can be prepared directly in situ with exogenous fibers. In this context, the exogenous fibers are prefabricated electrospun fibers and fibrils. In addition, in situ gelation creates a functional skin that serves as an ECM to facilitate fluid management, bacterial clearance, and wound healing.^[^ ^104^ ^]^ Wang et al. utilized polycaprolactone nanofibers, hyaluronic acid, and hyperbranched polyamide cross‐linking to fabricate a composite aerogel by freeze‐drying.^[^ ^105^ ^]^ This aerogel can collect wound exudates and transform them into a polycationic hydrogel that exhibits contact‐killing properties against pathogens and can actively scavenge reactive oxygen species in real time (Figure 5d). Owing to their precise and tunable molecular structures, metal‐organic frameworks (MOFs) have emerged as promising materials for antimicrobial applications. Wang et. al. obtained a viscous bacterial cellulose hydrogel loaded in situ with MOFs.^[^ ^106^ ^]^ This hydrogel provided sustained release of Zn^2+^, demonstrating its effective antibacterial properties.

With the advancement of antimicrobial and anti‐inflammatory FGs, the standards for functional material dressings have been raised. These dressings must exhibit extremely low leachability; be entirely free of antibiotics, metallic compounds, and nanoparticles; and leave minimal residue at the wound site upon removal. Wang et al. developed a self‐pumping oil‐water FGs dressing (SPD) using a 3D template‐induced wetting transfer (3D‐WET) polymerization strategy,^[^ ^26^ ^]^ which created aligned hydrated hydrogel channels. This FGs dressing consisted of a hydrophobic oil‐gel layer, a hydrophilic hydrogel layer, and aligned hydrated hydrogel channels. The synergistic effect of asymmetric wettability and aligned hydrated hydrogel channels endows the SPD with rapid and unidirectional drainage capabilities. By facilitating the removal of viscous fluids, SPD promotes the transformation of macrophages from the M1 to the M2 phenotype, thereby accelerating the healing of diabetic wounds. This research introduces a novel strategy for enhancing diabetic wound healing by managing viscous biological fluids rather than relying on pharmacological interventions.

The method of embedding drugs into fibers enhances the uniform dispersibility of the drugs and imparts controllable drug loading and release properties to FGs. The in situ gelation method for exogenous fibers is particularly suitable for hydrophilic drugs, enabling controlled release of the drugs by regulating the swelling behavior of the FGs. Both methods improved the antimicrobial and anti‐inflammatory properties of FGs to different drugs.

### 2.4 Stimulus Responsive

**Figure 6:**
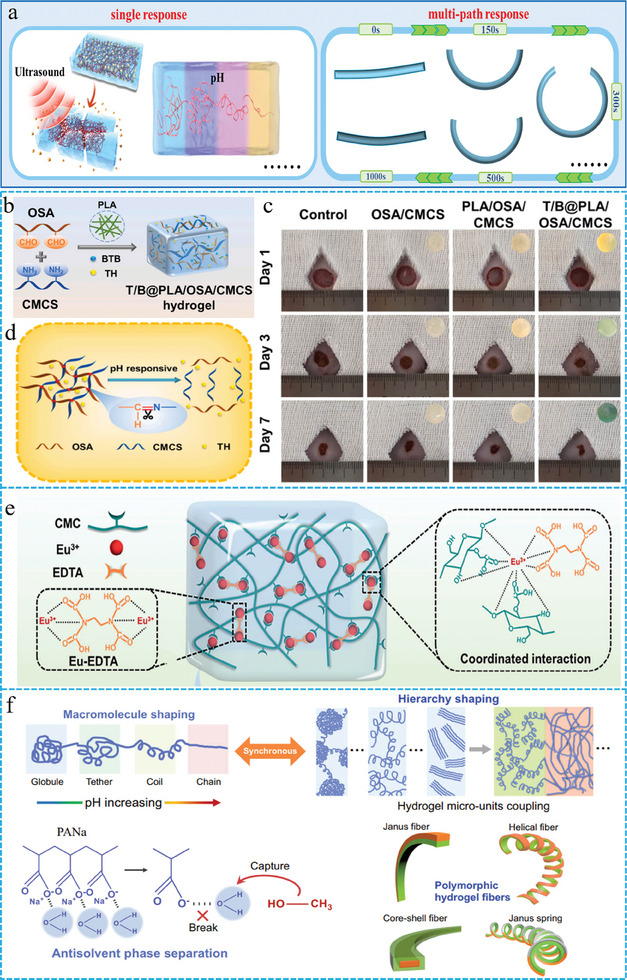
a) Schematic of two strategies for designing stimulus responsive. b) Schematic diagrams for the preparation route, c) pH‐responsive delivery about drugs and the wound healing d) of FGs. Reproduced with permission.^[^ ^111^ ^]^ Copyright 2024, Elsevier. e) Illustrations for the preparation hydrogel. Reproduced with permission.^[^ ^112^ ^]^ Copyright 2024, Wiley‐VCH. f) Schematic diagrams of FGs under different PH.^[^ ^113^ ^]^ Copyright 2024, Springer Nature.

Intelligently responsive FGs can rapidly respond to external stimuli, allowing them to maintain fundamental performance while possessing unique features such as visual monitoring, controllable drug release, shape transformation, and recovery. Currently, the external stimuli mainly include pH, electromagnetic fields, ultrasound, and photothermal effects.^[^ ^107^, ^108^, ^109^ ^]^ In terms of response, intelligent FGs have evolved from single responses to multipath responses. Here, we explore the latest breakthroughs of FGs from the perspective of the transition from single to multi‐pathway responses (**Figure** **6**a).

#### 2.4.1 Single Response

A single response refers to FGs making a specific behavioral change under an external stimulus. The latest breakthroughs in this area are mainly reflected in the addition of pH agents to monitor wound healing, as well as the integration of electrical, magnetic, and ultrasonic responsive agents to alter the composition and structure of FGs. First, the incorporation of pH‐responsive dyes or fluorescent complexes into the FGs system triggers a color change that enables visual monitoring of wound healing.^[^ ^110^ ^]^ Zhu et al. developed a pH‐responsive FGs dressing designed to monitor the pH of wounds through color changes and control drug release.^[^ ^111^ ^]^ The hydrogel matrix was formed by cross‐linking carboxymethyl chitosan (CMCS) and tetracycline hydrochloride (TH) onto an oxidized sodium alginate (OSA) framework by a Schiff base reaction (Figure 6b,c). Bromothymol blue (BTB), a pH‐sensitive dye, was used as a monitoring reagent (Figure 6d). Recently, Lu et al. developed self‐healing FGs with enhanced angiogenesis and real‐time monitoring capabilities, using a simple and effective strategy.^[^ ^112^ ^]^ This process involves the cross‐linking of carboxymethyl cellulose with a pre‐coordinated europium‐ethylenediaminetetraacetic acid (Eu‐EDTA) complex (Figure 6e). The fluorescence intensity of this hydrogel was associated with pH (4.5_–_7.5), allowing for real‐time measurement of pH levels to monitor wound status. This work provides guidelines for the application of molecularly regulated complexes. Recently, significant breakthroughs have been achieved in altering the state of FGs with pH changes. Ho et al. developed a method for controlling the mechanical properties of FGs through a macromolecular conformation‐shaping strategy.^[^ ^113^ ^]^ Using a pH‐dependent anti‐solvent phase separation technique, sodium polyacrylate polyelectrolyte macromolecules can be transformed from a coiled state to an extended state (Figure 6f). This approach significantly enhances the customizability of FGs, allowing the creation of various shapes.

**Figure 7:**
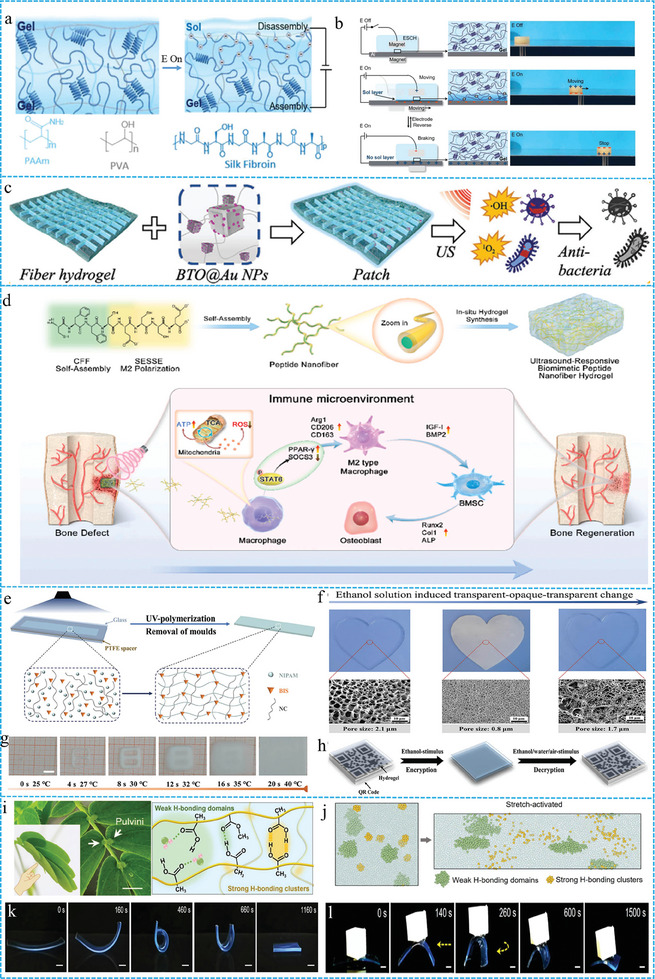
a) Diagrams of Electrical stimulation reversible sol layer. b) Demonstration about FGs movement under an electric field. Reproduced with permission.^[^ ^118^ ^]^ Copyright 2023, American Association for the Advancement of Science. c) The Schemes shows the composition and function of the patch.^[^ ^121^ ^]^ Copyright 2024, Wiley‐VCH. d) Schematic diagrams of self‐assembling and application peptide FGs. Reproduced with permission.^[^ ^53^ ^]^ Copyright 2024, Elsevier. e) UV‐induced polymerization and schematic structure of the NCPN hydrogels. f) The macroscopic and microscopic photographs of hydrogels by restricting the flow of solvents. g) The photos of hydrogels under different times and temperature. h) The encryption and decryption process of hydrogels. Reproduced with permission.^[^ ^126^ ^]^ Copyright 2022, Wiley‐VCH. i,j) The components and hydrogen bond clusters in fibers hydrogel. k,l) The deformation process of stress‐responsive fibers hydrogel. Reproduced with permission.^[^ ^27^ ^]^ Copyright 2024, Wiley‐VCH.

Subsequently, the functionality of FGs can be enriched by incorporating various responsive agents. For example, FGs exhibit reversible gelation ability upon the addition of electroresponsive or magnetoresponsive materials.^[^ ^114^, ^115^, ^116^, ^117^ ^]^ Song et al. pioneered the incorporation of silk fibroin supramolecular structures into covalent networks of polyacrylamide and polyvinyl alcohol polymers, culminating in the synthesis of electron‐responsive supramolecular covalent FGs.^[^ ^118^ ^]^ These FGs can emulate the dynamic secretion of mucus, with a reversible sol layer on their surfaces that responds to the application of an electric field (**Figure** **7**a). Inspired by mucosal mechanics, electro‐responsive supramolecular covalent FGs offer a promising avenue for regulating lubrication through electro‐active strategies (Figure 7b), thereby presenting significant potential in the design of soft actuators or robotics. As a non‐invasive energy source, ultrasound can activate FGs through extracorporeal manipulation without direct contact or chemical mediators.^[^ ^119^, ^120^ ^]^ Kong et al. developed an ultrasound‐responsive FGs patch that integrates both multifunctionality and biomimicry.^[^ ^121^ ^]^ They initially used electrospinning to create poly(ε‐caprolactone) square microfiber meshes. The subsequent integration of these fibers with the hydrogel precursor solutions resulted in the formation of FGs (Figure 7c). Furthermore, barium titanate decorated with gold nanoparticles (BTO@Au) was stimulated under ultrasound irradiation (US) to convert acoustic energy into electromechanical forces and generate reactive oxygen species (•OH, ^1^O_2_) to effectively eradicate bacteria. Recently, FGs have demonstrated excellent performance in the release of functional drugs under ultrasound stimulation, even in the absence of ultrasound sensitizers. Su et al. synthesized in situ calcium alginate FGs that integrated sodium alginate with assembled peptide nanofibers.^[^ ^53^ ^]^ Under ultrasonic stimulation, the disruption of the coordination between calcium ions and carboxyl groups led to hydrogel degradation, thereby facilitating the release of encapsulated peptide nanofibers (Figure 7d). This process activates mitochondrial glycolysis and the tricarboxylic acid cycle, thereby enhancing the polarization of M2 macrophages, and facilitating the creation of an immune microenvironment for bone regeneration.

#### 2.4.2 Multipath Response

Stress responses are common phenomena in multicellular organisms and are characterized by coordinated interactions between multiple cells and organs, leading to multipath and multistate responses to stimuli. However, the responses of current artificial materials are often confined to monotonous behaviors, with a lack of synergistic regulation among the various structural components.^[^ ^122^, ^123^, ^124^, ^125^ ^]^ The development of materials capable of multi‐pathway and multi‐state responses in biological systems has emerged as a major trend in the development of smart materials. Liu et al. developed a temperature/solvent multi‐responsive FGs (Figure 7e).^[^ ^126^ ^]^ This FGs exhibit exceptional mechanical properties, solvent‐induced high‐resolution reversible information recording, self‐encryption, and multiple decryption capabilities. Specifically, the clarity of the information recorded in the nanofiber structure was enhanced by restricting the flow of ethanol and other polar solvents, and the recorded information was erased either by wiping with water or by evaporating ethanol. This phenomenon is associated with changes in the pore size of FGs in polar solvents, such as ethanol and thermal stimuli, along with alterations in the chain state of the hydrogel. (Figure 7f) Therefore, this makes it possible to encrypt and decrypt information in an aqueous environment or under thermal stimulation (Figure 7g,h). Inspired by the mechanism of action in the mimosa, where the extensor and flexor cells in the petiole work in coordination, Wu et al. designed stress‐responsive FGs (Figure 7i).^[^ ^27^ ^]^ Upon the application of external stress, the strong hydrogen bond clusters of the FGs were broken, which facilitated the rapid diffusion of water molecules in the hydrogel. The weaker hydrogen bond cross‐linking structure tended to reduce the conformational entropy of the polymer chains, thereby promoting the elastic deformation recovery of the hydrogel (Figure 7j). Thus, when the FGs were immersed in water, they initially curled rapidly, and then gradually returned to their original state. This behavior is similar to the stress response of the mimosa when touched. In addition, during the deformation process, the coordinated changes in the differentiated hydrogen bond network enabled FGs to bear additional weight (Figure 7k,l). These properties are critical for the development of intelligent soft robots with autonomous movement capabilities.

In summary, intelligent hydrogels undergo physical or chemical changes in response to external stimuli that affect their characteristics, such as shape and volume. The incorporation of nanofibers enhances the mechanical properties of intelligent hydrogels and facilitates the simulation of complex fibrous structures within biological organisms. Through the synergistic regulation of various structural components, intelligent FGs can achieve multi‐pathway and multi‐state stress responses within biological systems. By integrating different materials and employing diverse stimuli, it is possible to achieve complex programming steps within a single system, thereby enriching the multifunctionality of FGs. Therefore, the current trend in the development of intelligent FGs is moving towards composite and multifunctional integration.

### 2.5 ECM‐Like Structure

**Figure 8:**
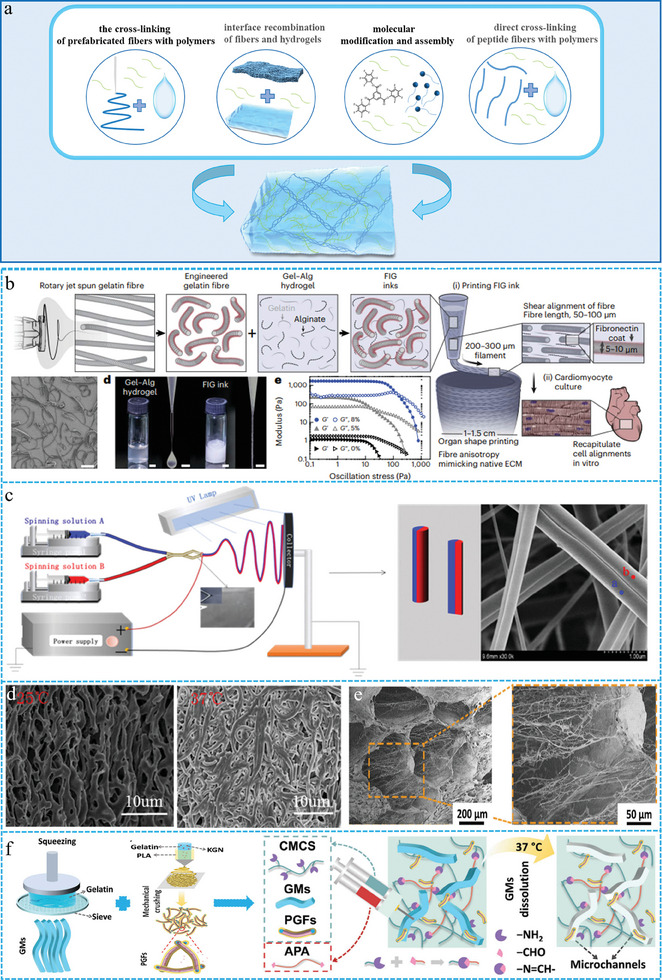
a) Schematic of four strategies for the construction ECM‐like structures. b) The preparation process, SEM images and performance tests of 3D‐printed tissue scaffolds. Reproduced with permission.^[^ ^136^ ^]^ Copyright 2023, Springer Nature. c,d) The s‐b‐s electrospinning process and SEM images of Janus hydrogel fibers. Reproduced with permission.^[^ ^137^ ^]^ Copyright 2024, Royal Society of Chemistry. The preparation process f) and SEM images e) of APA/CMCS/KGN @ PGF/GM hydrogels. Reproduced with permission.^[^ ^138^ ^]^ Copyright 2023, Wiley‐VCH.

The native ECM possesses a heterogeneous and multi‐hierarchical complex structure, whose kinetics and fiber nanostructures can provide cells with a stable and dynamic biophysical environment as well as biochemical signals, and guide cell behavior.^[^ ^127^, ^128^ ^]^ The simulation and reconstruction of the structure and function of ECM is has become an important direction of research.^[^ ^129^ ^]^ Due to their easily adjustable physicochemical properties and dynamic networks that accommodate cell survival, FGs can encapsulate and influence cells. On the other hand, FGs can enhance cell‐matrix and cell‐cell interactions, which in turn significantly improve the mechanotransduction, metabolic energy, and biological action of encapsulated stem cells. These characteristics make them unique biomaterials for 3D cell culture, in vitro modeling, or TE applications.^[^ ^130^, ^131^ ^]^ At present, the design of functional FGs with natural ECM properties remains the focus of biomimetic hydrogel research. The four strategies for constructing biomimetic FGs with ECM‐like structures are as follows (**Figure** **8**a): cross‐linking of prefabricated fibers with polymers, interfacial recombination of fibers and hydrogels, molecular modification and assembly, and direct cross‐linking of peptide fibers with polymers.

#### 2.5.1 The Cross‐Linking of Prefabricated Fibers with Polymers

In this method, prefabricated fibers are obtained through electrospinning, wet spinning, and stretching, which are then incorporated into hydrogel systems via physical interactions.^[^ ^132^, ^133^ ^]^ In contrast to the FGs formed by chemical reactions, this approach does not require chemical agents, resulting in improved biocompatibility.^[^ ^134^, ^135^ ^]^ Therefore, these hydrogels have significant potential for biomedical applications. Recently, breakthroughs have been made in obtaining prefabricated fibers through rotary spinning, side‐by‐side spinning, and core sheath coaxial spinning. Parker et al. developed a hydrogel ink containing prefabricated gelatin fibers that was used to print 3D organ scaffolds.^[^ ^136^ ^]^ Gelatin fibers were prefabricated by rotary jet spinning, and the preformed fibers were cut by ultrasonication. It was then mixed with gelatin‐alginate gel to obtain the hydrogel ink. During 3D printing, fiber cross‐linking generates an ECM‐like structure under shear stress (Figure 8b). The injected gelatin fiber acted as a rheological modifier in the hydrogel ink, printing complex 3D objects without a sacrificial bath. In addition, these fibers provided biochemical and microstructural clues, promoting cell adhesion and self‐organization to form functional syncytial bodies. Zha et al. developed a biomimetic ECM hydrogel containing Janus fibers using side‐by‐side electrospinning combined with UV irradiation. (Figure 8c) Owing to the self‐coiling properties of Janus fibers at body temperature, the tight entanglement between Janus fibers and the hydrophobic interactions between them result in an ECM‐like structure and the sol‐gel transition when the temperature reaches 37 °C. (Figure 8d) This FG exhibited excellent stability, robustness, elasticity, and self‐healing ability under physiological conditions.^[^ ^137^ ^]^ Yan et al. prepared prefabricated fibers by coaxial electrospinning followed by incorporation of a hydrogel matrix to construct injectable FGs. The introduction of nanofibers enhanced the mechanical properties of the hydrogel and simulated the fibrous and porous structure of natural ECM (Figure 8e,f).^[^ ^138^ ^]^ FGs facilitate the inward growth of cells in the internal pore structures of the hydrogels.

#### 2.5.2 Interface Recombination of Fibers and Hydrogels

**Figure 9:**
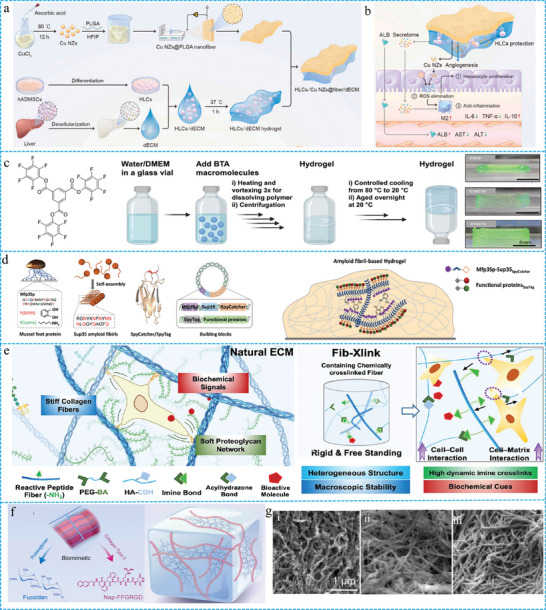
a) Schematic diagrams for design strategy and cells delivery b) of the FGs. Reproduced with permission.^[^ ^45^ ^]^ Copyright 2023, Elsevier BV. c) Diagrams for supramolecular assembly and multiple layer structures of C_8_C_8_, C_10_C_10_ and C_12_C_12_ were printed. Reproduced with permission.^[^ ^142^ ^]^ Copyright 2023, American Association for the Advancement of Science. d) Schematic representation of protein assembly. Reproduced with permission.^[^ ^130^ ^]^ Copyright 2023, Wiley‐VCH. e) The design strategy diagram for FGs. Reproduced with permission.^[^ ^151^ ^]^ Copyright 2023, American Chemical Society. f,g) The components and SEM images of the hydrogel. Reproduced with permission.^[^ ^146^ ^]^ Copyright 2024, Elsevier.

The prefabricated fibers and hydrogels can also be constructed as FGs with ECM structures by interface recombination.^[^ ^139^ ^]^ Despite the limited amount of existing research on this type of FGs, it exhibits distinctive characteristics and holds significant potential for clinical applications. These two systems are suitable for use in acellular matrix (dECM) hydrogels. Recently, Li et al. developed a multifunctional biomimetic platform through an interfacial composite of poly (lactic‐co‐glycolic acid) nanofibers loaded with Cu‐based nanoenzymes and liver dECM hydrogels (**Figure** **9**a).^[^ ^45^ ^]^ This platform features a biomimetic structure designed for the in situ delivery of functional hepatocyte‐like cells derived from human mesenchymal stem cells to effectively treat acute liver failure (Figure 9b).

#### 2.5.3 Molecular Modification and Assembly

The ECM is formed through directional interactions between small and large molecules that result in transient supramolecular fibers. This process involves various biophysical and chemical signals that regulate cellular behavior, thereby playing a crucial role in tissue growth and regeneration. These structures can be modulated through molecular modifications and subsequently assembled into FGs with ECM‐like structures. The molecules included supramolecular and natural protein molecules. Supramolecular FGs are dynamic hydrogels assembled from molecular modules through directional and non‐covalent interactions.^[^ ^140^, ^141^ ^]^ The reversibility of non‐covalent interactions enhances the adaptability of hydrogels. This adaptability is conducive for cell diffusion and migration within the hydrogel, which does not require degradation and has a large pore size. By modulating the hydrophobic substituents of supramolecular benzene‐1, 3, 5‐tricarboxamide (BTA), Baker et al. regulated of the ECM‐like structure and viscoelastic properties of FGs Specifically, a small pool of BTA FGs was created by changing the length of the end base carbon from 6 to 12 carbon units (Figure 9c).^[^ ^142^ ^]^ This supramolecular design strategy realizes precise control of the mechanical properties of FGs and has good universality and scalability, providing new ideas for tissue engineering and cell biology research. Compared with traditional organic supramolecular, natural protein macromolecules possess good biocompatibility, biodegradability, and genetic modifiability, which are suitable as basic units for constructing protein functional materials.^[^ ^143^, ^144^ ^]^ As natural protein molecules, amyloid proteins can be regulated to produce fibrous structures, which are then assembled into ECM‐like FGs. a fiber structure and then assemble into ECM‐like FGs.^[^ ^145^ ^]^ Inspired by the natural self‐assembled single proteins, Ge et al. developed temperature‐induced programmable FGs with ECM‐like structures using genetic module fusion (Figure 9d).^[^ ^130^ ^]^ These FGs not only fill the gap in the field of ternary protein fusion but also exhibit excellent adhesion, high stability, and broad applicability.

#### 2.5.4 Direct Cross‐Linking of Peptide Fibers with Polymers

An ECM with a heterogeneous and hierarchical structure is formed through the structural stability provided by fibrin and the viscoelasticity generated by interfibrous matrix substances. Specifically, some rigid fibrous ECM components such as collagen can promote macroscopic stability to ensure the integrity of the tissue.^[^ ^146^ ^]^ And some fibrous ECM components, such as fibronectin, can effectively provide signaling molecules to the resident cells.^[^ ^147^ ^]^ Loosely packed inter‐branchial polysaccharides and proteoglycans provide microstructural dynamics to accommodate cellular activities including drastic shape changes such as diffusion, migration, and proliferation.^[^ ^148^, ^149^ ^]^ Hetero‐composite hydrogels containing biomimetic fiber bundle components can potentially mimic multiple biophysical and biochemical functions of the native ECM, thereby providing the desired cellular adaptation and inducible synthetic 3D matrix to promote tissue regeneration.^[^ ^150^ ^]^ Bian et al. designed and developed B‐polypeptide fiber‐hyaluronic acid FGs, in which the peptide fibers with a hard β‐sheet layer enhanced the macroscopic stability of the FGs,^[^ ^151^ ^]^ while the dynamic imide bonding between the peptide fiber and the polymer network advanced the micro‐dynamic properties of the hydrogel (Figure 9e). ECM‐like FGs have a dynamic network that can adapt to cell behavior in a timely manner and enhance the interaction between cells and the matrix, thereby promoting the osteogenic differentiation of stem cells through the coupling of mechanical signal transduction and energy metabolism. Recently, inspired by the random interlacing structure of proteoglycan and collagen in natural cartilage, Zhu et al. prepared a new glycopeptide hydrogel (Figure 9f) based on self‐assembled polypeptides and marine polysaccharides to realize the bionics of the collagen and polysaccharide components of cartilage.^[^ ^146^ ^]^ This hydrogel was composed of a fiber‐interwoven structure, similar to that of natural cartilage, through intermolecular self‐assembly reactions (Figure 9g). In addition, the hydrogel regulated tissue regeneration and oxidative stress by promoting cartilage ECM secretion and eliminating excess ROS.

These four methods each have their advantages, and their purpose was the same to prepare FGs with an ECM‐like structure. These methods can replicate fibrous structures and effectively transfer biophysical and biochemical properties, thereby facilitating the study of cell‐matrix interactions. The accurate replication of the appearance and specialized functions of biological tissues has advanced the development of next‐generation biomimetic materials.

## 3 Application

### 3.1 Sensors

**Figure 10:**
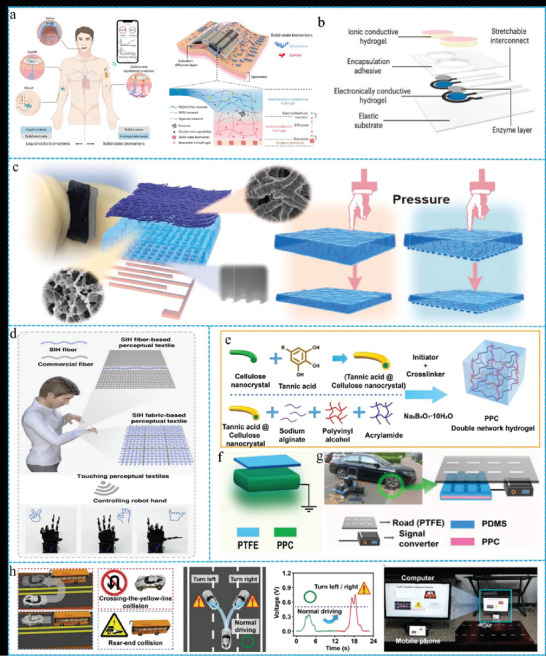
a) Schematics of producing and detecting body fluids by SEB sensor in the human body. b) Schematics of the SEB sensor structure. Reproduced with permission.^[^ ^153^ ^]^ Copyright 2024, Springer Nature. c) Schematics of the assembled piezoresistive sensor. Reproduced with permission.^[^ ^159^ ^]^ Copyright 2024, Elsevier. d) Schematics for the gestures of a robot by man touching the SIH‐based textiles. Reproduced with permission.^[^ ^88^ ^]^ Copyright 2024, Springer Nature. e) The preparation process of PPC hydrogel and f) schematics of TENG sensor. g,h) Schematics shows that the detection and processing of the driver state and the vehicle state. Reproduced with permission.^[^ ^164^ ^]^ Copyright 2023, Wiley‐VCH.

Sensors can convert external stimuli into detectable digital electrical signals to accurately perceive environmental stimuli and show significant vitality in areas such as human motion detection, human‐machine interfaces, and smart textiles.^[^ ^152^ ^]^ Liu et al. developed a dual‐layer ion‐electron hydrogel that functions as a sensor capable of the in situ detection of ubiquitous solid‐state biomarkers on human skin (**Figure** **10**a,b).^[^ ^153^ ^]^ This sensor exhibits the capacity for continuous monitoring of both water‐soluble and insoluble solid analytes and is characterized by an exceptionally low detection threshold. As novel conductive materials, CFGs possess unique structural characteristics, strong mechanical flexibility, and easy processing, which have facilitated the development of flexible sensors.^[^ ^154^, ^155^ ^]^ Despite the numerous designs of CFGs that have been developed, several challenges still remain before their practical application. Initially, it encountered a variety of challenges in practical applications, such as extremely cold conditions.^[^ ^156^ ^]^ Therefore, there is a need to develop CFGs with antifreeze, moisturizing properties and high mechanical performance. Current improvement strategies involve the introduction of small molecule antifreeze moisturizers, including organic solvents (glycerol, ethylene glycol, and dimethyl sulfoxide) and inorganic salts (sodium sulfate and lithium chloride).^[^ ^54^, ^157^ ^]^ Although these additions enhance the environmental resistance of hydrogels, the introduction of small molecules complicates the preparation process and negatively impacts the overall integration. Importantly, these small molecule formulations tend to migrate in water. These drawbacks limit the long‐term stability and applicability of hydrogels in various applications. This research demonstrates that establishing strong interactions between the polymer chains of hydrogels and water molecules significantly enhances the antifreeze and moisturizing properties of hydrogels.^[^ ^158^ ^]^

In addition, electronic textiles serve as a bridge between humans and electronic devices, providing functionalities such as communication, sensing, and display. However, electronic textiles currently face the challenge of achieving precise tactile localization by humans and supporting hazard identification and response capabilities. CFGs have achieved breakthroughs in smart wearable devices. Recently, Jiang et al. integrated lightweight nanofilms with highly elastic hydrogels to create a dual‐module film‐hydrogel structure.^[^ ^159^ ^]^ This module amalgamates the functionalities of thermal energy collection, solar power, and piezoresistive sensing, culminating in the development of a trienergetic, portable mini‐sensor that operates independently of electrical power (Figure 10c). Zhang et al. developed an intelligent sensing textile based on conductive ion hydrogel fibers (SIH), that can generate electrical responses to external hazards and precisely detect human touch (Figure 10d).^[^ ^88^ ^]^ Now, triboelectric nanogenerators (TENGs) have emerged as a popular research topic because of their excellent sensitivity, convenience, ease of fabrication, lightweight, seamless integration with other electronic components, and self‐powering capabilities.^[^ ^160^, ^161^ ^]^ With the rapid rise of 5G networks and the Internet of Things, several smart transportation monitoring devices based on TENGs have been developed.^[^ ^162^, ^163^ ^]^ However, challenges such as preventing drowsy driving, ineffective traffic accident management, and a lack of self‐healing capabilities persist. Yang et al. prepared a novel PVA‐PAM/tannic acid‐modified cellulose nanocrystal double‐network hydrogel (PPC) (Figure 10e).^[^ ^164^ ^]^ Subsequently, PPC was embedded in polydimethylsiloxane (PDMS) to fabricate a single‐electrode TENG sensor (Figure 10f). Finally, multiple TENG sensors (Figure 10g) were integrated to construct a self‐powered intelligent traffic monitoring system based on the piezoresistive effect for driver state detection and the friction‐electrification effect for vehicle state monitoring. This system comprises flexible wearable PPC strain sensors and PPC‐based TENG sensors (Figure 10h), and innovatively enables the simultaneous monitoring of driver and vehicle states to ensure traffic safety and facilitate effective accident management, which has significant implications for the future development of smart city traffic.

In summary, sensors based on CFGs are primarily used in human motion detection and smart wearable sectors. The developmental characteristics of these sensors in domains are as follows: Functionally, CFGs sensors have evolved from single‐mode detection to multi‐output capabilities, progressively aligning with accurate and complex biomimetic applications. Their adaptability extends from ambient to extreme conditions, meeting the demands of specialized environments and broadening their scope of application. The stability is improving with ongoing efforts to mitigate the contradictions between water evaporation and performance. With the continuous expansion of application fields, sensors based on FGs are emerging in intelligent transportation systems. It is assumed that sensors based on FGs will gradually be achieved through scientific efforts.

### 3.2 Dressing

**Figure 11:**
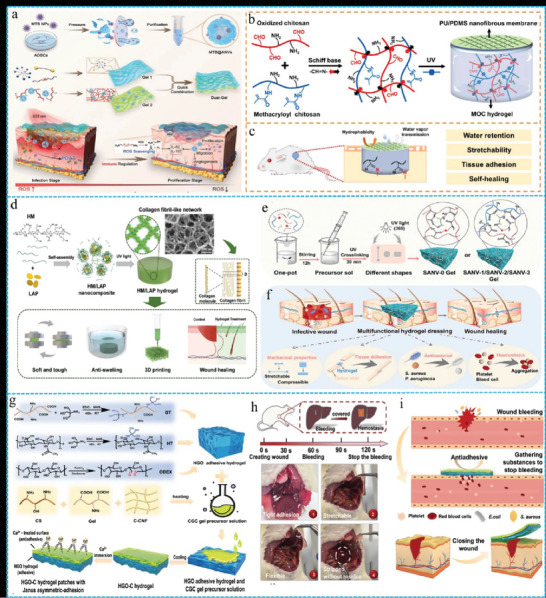
a) Schematic diagram of the preparation process of Dual‐Gel and application for wound healing. Reproduced with permission.^[^ ^168^ ^]^ Copyright 2024, Wiley‐VCH. b,c) the preparation process of composite material and application for wound healing. Reproduced with permission.^[^ ^169^ ^]^ Copyright 2023, Elsevier. d) The preparation and properties of the composite hydrogel. Reproduced with permission.^[^ ^175^ ^]^ Copyright 2024, Elsevier. e,f) The preparation process of hydrogel and application for wound healing. Reproduced with permission.^[^ ^176^ ^]^ Copyright 2024, Elsevier. g) Schematic diagram of the preparation process of HGO‐C hydrogel. h,i) The photos and diagrams in emergency hemostasis and promoting wound healing in rat liver. Reproduced with permission.^[^ ^177^ ^]^ Copyright 2024, Wiley‐VCH.

Wounds are one of the most prevalent clinical problems, and there are various dressings which can help with wounding healing.^[^ ^165^ ^]^ These dressings are designed to manage different aspects of the healing process, such as moisture retention, protection against infection and promotion of tissue regeneration.^[^ ^95^, ^166^, ^167^ ^]^ Hydrogels have emerged as one of the most promising dressings to create a moist environment for wound healing. Due to unique structures and properties, FGs are increasingly becoming focal materials for wound dressings, particularly for chronic wounds and burns. Liao et al. engineered a dual‐layer hydrogel for burn wound healing, which promotes comprehensive wound tissue repair through programmed regulation of ROS.^[^ ^168^ ^]^ The inner layer of the hydrogel (Gel 2) initially responded to bacterial hyaluronidase by deploying stem cell‐derived nanovesicles under light exposure, generating ROS to eradicate bacteria. The outer layer (Gel 1) persistently consumed the excess ROS at the wound site, thereby facilitating accelerated tissue regeneration (**Figure** **11**a).

Currently, the practical application of FGs is constrained by their extensibility as well as issues such as water retention barriers, lack of robust self‐healing capabilities, and unstable adhesion to the wound bed. Although the introduction of glycerol or salts into hydrogels can enhance water retention, it also reduces their biocompatibility. Therefore, developing FGs composite materials that can maintain long‐term moisturization, stretchability, tissue adhesion, and self‐healing properties remains a significant challenge. Ding et al. obtained a breathable and hydrophobic nanofiber membrane embedded with polyurethane (PU) in polydimethylsiloxane (PDMS) by electrospinning, which served as a water‐retaining and breathable upper support layer.^[^ ^169^ ^]^ A dual‐crosslinked hydrogel composed of methacrylated chitosan (MC) and oxidized chitosan (OC) was used as the lower layer in contact with the wound surface. This composite material exhibited enhanced water retention (Figure 11b), superior tensile properties, rapid self‐healing, and excellent adhesive abilities. It also demonstrated an effective therapeutic action in a full‐thickness rat skin wound model. (Figure 11c)

Although there have been reports of FGs with good mechanical properties and fibrous structures, most of them are not injectable because of their uncontrollably high cross‐linking and self‐assembly.^[^ ^170^, ^171^, ^172^ ^]^ Furthermore, sheet‐like FGs dressings can lead to incomplete coverage of irregular wounds and inadequate filling of deep wounds. Injectable hydrogels offer significant advantages over other hydrogels, such as complete defect filling, less discomfort for patients, and a lower risk of infection.^[^ ^173^, ^174^ ^]^ Therefore, the development of injectable FGs can further advance their practical applications. Wang et al. used a physical blend of methacrylated hydroxypropyl chitosan (HM) and lithium montmorillonite (LAP) to autonomously form HML nanocomposites.^[^ ^175^ ^]^ Under ultraviolet irradiation, the double bonds in the nanoparticles undergo chemical cross‐linking, creating a 3D interwoven network of nanofibers to form FGs. Then, 3D printing achieved customized shapes of complex biological structures (Figure 11d). Data from mice demonstrated its potential as a personalized wound dressing. This work offers a general strategy for the development of novel biomimetic FGs as personalized wound dressings.

As FGs become more thoroughly studied in the context of wound dressings, there are still significant challenges to their effective transition them to practical applications: i) How to mass‐produce hydrogel dressings that are mechanically robust and multifunctional even when swollen and mimic the microenvironment of tissues and are functionally diverse. This involves the development of methods that combine high performance and environmental sustainability. ii) Reducing the costs of raw materials and production. For example, the production of bacterial cellulose is limited by factors such as low production rates of bacterial strains, expensive fermentation processes, and high downstream processing costs, which hinder its mass production. To address these challenges, Wang et al. developed multifunctional FGs based on sodium alginate (SANV) through a simple one‐pot method and UV light‐induced cross‐linking polymerization to effectively promote wound healing (Figure 11e).^[^ ^176^ ^]^ Once mixed, the precursor solution was easy to transport and can be prepared into dressing samples on‐demand, conforming to the irregular shapes of the wounds at any time (Figure 11f). To realize the application of FGs dressings in human medicine, research has progressed from mouse and rat wound models to pig models. Transitioning to larger animal models that closely mimic human physiology is essential to validate the effectiveness and safety of dressings before conducting clinical trials in humans. Zhang et al. developed a hydrogel (HGO) featuring asymmetric adhesive properties with an adhesive side and a non‐adhesive side.^[^ ^177^ ^]^ HGO was cross‐linked through reactions involving oxidized dextran (ODEX) with modified hyaluronic acid (HT) and modified gelatin (GT). CGC consist of chitosan (CS), gelatin (Gel), and a water dispersion of carboxylated cellulose nanofibers (C‐CNF). By coating the CGC solution atop the HGO and performing a simple CaCl_2_ soaking treatment, asymmetric adhesive characteristics were acquired (Figure 11g). These Janus asymmetric adhesive FGs demonstrated efficacy in emergency hemostasis and promotion of wound healing in rat liver (Figure 11h) and skin injury models (Figure 11i).

As ideal materials for wound dressings, FGs have evolved from single functions to integrated multifunctional capabilities, such as antibacterial, hemostatic, and immunomodulatory. Moreover, its mechanical properties including anti‐swelling, tensile strength and elasticity are continuously enhanced. Despite reports of FGs dressings with multifunctional properties and appropriate mechanical properties even when swollen, there are significant challenges in the way of their actual clinical application. These include developing large‐scale, environmentally friendly synthesis methods, creating simple and efficient preparation devices, reducing the costs of materials and production, and importantly, ensuring safety and clinical application in large mammals and humans. Further investigation and optimization are required in areas such as adhesive performance, drug delivery strategies, and in vivo degradation pathways. Overall, FGs provide hope for the management of difficult‐to‐heal wounds, particularly chronic wounds and burns, and offer significant potential for improving outcomes in these challenging areas.

### 3.3 Tissue Scaffolds

**Figure 12:**
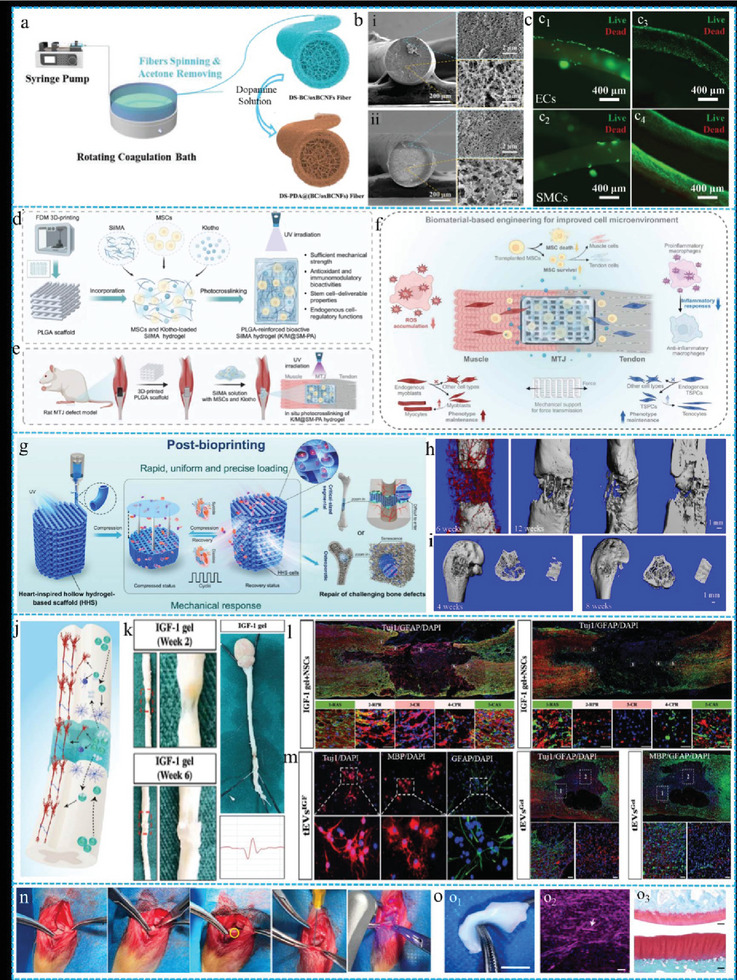
a,b) Schematic diagram of the preparation process and SEM images of FGs. c) The photos about cells culture with FGs. Reproduced with permission.^[^ ^180^ ^]^ Copyright 2024, Wiley‐VCH. d) The preparation process of hydrogel. e,f) Illustrations of the MTJ defect process and mechanism in rats of the hydrogel. Reproduced with permission.^[^ ^182^ ^]^ Copyright 2024, American Association for the Advancement of Science. g) Schematic diagrams of the post‐bioprinting strategy and application for repair bone defects. h,i) (µCT) images of bone and blood vessels after surgery. Reproduced with permission.^[^ ^183^ ^]^ Copyright 2024, Springer Nature. j) Illustrations of IGF‐1 gels with NSCs for promoting the repair of neurons and myelin sheaths. k) The SCI model with the implantation of IGF‐1 gels and electrical signaling transmission in SC. l,m) Double immunostaining in the IGF‐1+NSC groups and tEVs Gel. Reproduced with permission.^[^ ^186^ ^]^ Copyright 2024, Wiley‐ VCH. n) The process of meniscus tears modeling and adhesives heal. o) Macrograph, H&E staining and immunofluorescence of repaired meniscus. Reproduced with permission.^[^ ^187^ ^]^ Copyright 2024, Springer Nature.

The ECM constitutes a 3D network of biomolecules comprising non‐cellular components within tissues and organs. The nanofibrous network structure of the ECM plays a pivotal role in the formation and maintenance of the physical structures of cells and tissues. For instance, the fibrous organization of the ECM facilitates long‐distance intercellular communication, and a contraction‐induced strain within one cell can propagate along the ECM fibers to a distant cell, subsequently translating into a biochemical signal. Furthermore, the mechanical state of the ECM contributes to the regulation of various cellular functions, including contraction, migration, proliferation, and activation. Consequently, the ECM is crucial in guiding cellular growth. However, challenges such as limited natural ECM sources, the risk of cross‐species viral transmission, inferior mechanical performance, and structural stability persist. Biomimetic hydrogels with fibrous structures surpass non‐fibrous hydrogels in terms of cellular proliferation, migration, and spreading. Liu et al. successfully facilitated the continuous fabrication of hollow fibrous BC‐based biological scaffolds (**Figure** **12**a)^[^^180]^ using a microfluidic coaxial spinning apparatus in conjunction with a low‐speed rotating coagulation bath spinning technique. Subsequently, the author achieved two bio‐scaffolds with dense outside and sparse inside heterogeneous structures (DS‐BC/oxBCNFs and a surface deposition of polydopamine, DS‐PDA@(BC/oxBCNFs) (Figure 12b). Due to the densification of the surface, FGs have experienced enhancements in strength, modulus, and extensibility. Moreover, the DS‐PDA@(BC/oxBCNFs) FGs can adhere to polygonal endothelial cells (ECs) and spindle‐shaped smooth muscle cells (SMCs), characterized by transverse dimensions, and can completely cover the fiber surface (Figure 12c3,c4). In contrast, the DS‐BC/oxBCNFs FGs adhered to oval‐shaped cells of both types and in limited quantities (Figure 12c1,c2), suggestions that the DS‐PDA@(BC/oxBCNFs) FGs exhibited superior cell adhesiveness. This is because the positively charged amino groups in PDA reduce the negative surface potential of the macrofibres, whereas controlled removal of the solvent densifies the surface of the macrofibres, thereby increasing the contact area between the cells and fiber surface.

Because of the inadequate blood supply and sluggish metabolism of chondrocytes, self‐healing of articular cartilage after injury is quite difficult. Currently, surgical treatments for cartilage damage, such as microfracture surgery, autologous and allogeneic cartilage transplantation, are hampered by shortcomings, including scarcity of suitable donors, difficulty in restoring the original structure and physiological functions, and subsequent cartilage degeneration.^[^ ^181^ ^]^ Due to minimally invasive nature, adaptability to irregularly shaped defects, and ease of loading with cells and active pharmaceuticals, injectable hydrogels have gained widespread application in cartilage regeneration. Zhang et al. developed 3D‐printed bioactive FGs containing mesenchymal stem cells and Klotho. The well‐organized structure of the poly(lactic‐co‐glycolic acid) (PLGA) scaffold provides sufficient mechanical strength to support the physiological functions of MTJ.^[^ ^182^ ^]^ The incorporation of mesenchymal stem cells enhances the regenerative bioactivity of muscles and tendons, while the addition of klotho improves the pathological environment for both exogenous stem cells and endogenous MTJ‐resident cells following MTJ injury. The integration of the photo‐crosslinked silk fibroin methacrylate hydrogel into the PLGA scaffold created a 3D hydrated microenvironment that supported the retention and survival of mesenchymal stem cells (Figure 12d). After implantation into a rat MTJ defect model (Figure 12e), all the experimental results demonstrate that the FGs system enhanced the structural restoration of muscle, tendon, and the musculotendinous interface, and improves the functional recovery of the injured MTJ. This efficacy is attributed to its immunomodulatory and antioxidative properties, which improve the microenvironment after tendon injury, regulate the behavior of both exogenous and endogenous cells, and promote healing (Figure 12f). Bone defects often result in restricted migration or diminished regenerative capabilities of endogenous cells, making self‐healing challenging. Although significant advancements in bone repair have been made over the past two decades, difficulties persist, including time‐consuming ex vivo cultivation and the inability to precisely control cell distribution. 3D printing can realize accurate cell delivery; however, the activity of cells and mechanical stability of the stent pose challenges during the printing process. Inspired by the beating heart pumping of blood, Ruan et al. proposed a new strategy of mechanically assisted “3D bioprinting plus+”.^[^ ^183^ ^]^ First, a cell‐loaded hollow FGs scaffold with mechanical response was obtained using methylacrylylated gelatin/lithium silicate nanoclay/N‐acrylylglycine as a biological ink combined with 3D printing (Figure 12g). Micro‐computed tomography (µ CT) reconstructed images of the scaffold showed that neovascularization increased in the bone defect at 6 weeks. Tubular and rod‐shaped bridging defect sites with new bone formation were observed at 12 weeks (Figure 12h). In addition, the reconstructed images of osteoporotic bone defects in rats showed that new bone increased in the cancellous bone defect during 4−8 weeks (Figure 12i). These results highlighted the effectiveness of FGs scaffolds in bone repair and functional reconstruction. This strategy effectively solves the contradiction between cell viability and mechanical stability of scaffolds in the current extrusion 3D bioprinting process, and provides new ideas for tissue engineering and regenerative medicine.

Spinal cord injury (SCI) is a permanent motor and sensory dysfunction of the spinal cord caused by strong mechanical forces that disrupt the bidirectional communication of basic neurological functions between brain and spinal cord. Neural stem cell (NSCs) transplantation has brought hope for the treatment of SCI, but there are still some problems resulting in limited therapeutic effects, such as low survival rate of the transplanted cells, difficulty in enriching the injured site, and differentiation into neurons and oligodendrocytes.^[^ ^184^, ^185^ ^]^ FGs are ideal microenvironments for culturing NSCs in vitro. Shen et al. synthesized a supramolecular FGs by adding a self‐assembling peptide to the N‐terminus of the C domain from insulin‐like growth factor‐1 (IGF‐1).^[^ ^186^ ^]^ The implanted FGs connected the neurons of SCI neuron fibers and promoted myelin regeneration (Figure 12j). The SCI view showed that lesions in the head and tail appeared as new links at 2 weeks, the head and tail were almost fully connected, and electrical signal transmission was restored at 6 weeks (Figure 12k). In addition, the results of Tuj1 and GFAP double staining showed that SCI of the implanted NSCs hydrogels appeared in Tuj1 positive cells as well as nerve fibers from astrocyte boundaries extended to the cavity, and then connected the ends of the damage. These results proved that it could promote the survival, proliferation and differentiation of NSCs into neurons and oligodendrocytes (Figure 12l). IGF‐1 gel immuno staining results showed that the number of neurons and oligodendrocytes increased, and the growth of EVs extended the neural processes (Figure 12m). These data suggested that FGs can significantly promote neurite outgrowth and remyelination at injury sites, thereby improving neural recovery after SCI. This study offers a new way to develop FGs with stem cell therapy and has opened up a new direction for the treatment of SCI. These facts indicate that FGs can promote the corresponding organization, reconstruction, and restoration as they act as cell carriers. Recently, the use of FGs without cells has been a major breakthrough on the reconstruction and restoration of biological tissues. Ouyang et al. developed a silk FGs consisting of methacrylated silk cellulose, phenylborate ions liquid, and the growth factor (TGF‐β1).^[^ ^187^ ^]^ Phenylborate ion liquids enhance the interactions between supramolecular and hydrogen bonds, thereby improving the wet adhesion, swelling resistance and fatigue resistance of the hydrogel. The results of the relevant staining of the rabbit meniscal injury (Figure 12n) showed that the FGs achieved seamless repair and dense reconstruction of the torn meniscus (Figure 12o). This study provides a promising and revolutionary strategy for repairing meniscal tears.

FGs serve as excellent media for in vitro cell culture, creating an environment conducive to cell proliferation and differentiation. FGs have extensive applications in biological tissue scaffolds, from initial cell adhesion and proliferation to the carrier of stem cells, leading to the reconstruction and restoration of bone, cartilage, tendons, and spinal cord in biological tissues. In addition, it can be used as a biological scaffold to repair the meniscus and pelvic floor muscles. FGs are promising for tissue engineering applications, however, their integration into clinical practice and large‐scale production necessitates further optimization of the mechanical stability and cellular activity within the scaffold. The development of advanced bionic FGs requires a nuanced understanding of the interplay between the structure and function of the ECM. However, it is necessary to reach a balance between the mechanical stability of the scaffold and the cell viability of FGs, and to explore the mechanism between the structure and performance of the ECM for its real clinical application and mass production. We look forward to developing further ideal bionic FGs.

### 3.4 Others

**Figure 13:**
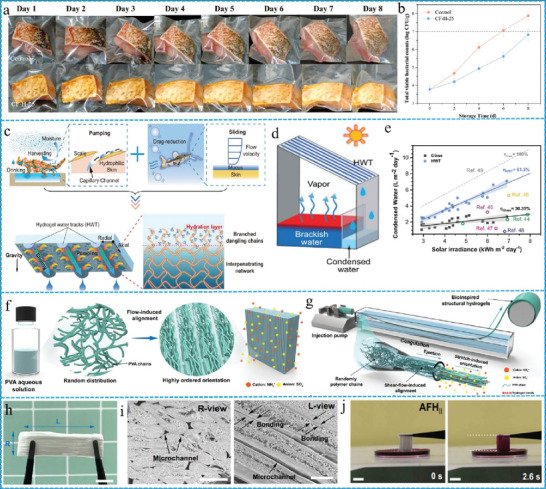
a,b) Fish fillet packaging experiment between control and CF‐H‐25 group at different days. Reproduced with permission.^[^ ^188^ ^]^ Copyright 2024, Elsevier. c) Diagrams of continuous water capture and directional water movement. d) Schematic diagram of condensed water collection. e) Comparative data of condensed water collection between HWTs and others. Reproduced with permission.^[^ ^189^ ^]^ Copyright 2023, Oxford University Press. f,g) Schematic of fluid‐induced techniques and short‐distance coagulation bath technique. h) The photos of fibrous hydrogels. i) SEM images of the fibrous hydrogels under different directions. j) The photos of the unidirectional water transport. Reproduced with permission.^[^ ^190^ ^]^ Copyright 2024, Springer Nature.

Food packaging technology plays an indispensable role in the food supply chain as it ensures the maintenance of food quality, freshness and nutritional value, and effectively extends shelf life. This technology serves as a barrier against environmental factors and as a critical medium for preserving the integrity and sustainability of food products throughout its distribution. FGs, with high‐water content, flexibility, and 3D porous structures can absorb significant amounts of water. This functionality enables them to regulate moisture within food packaging and effectively absorb excess water produced by the packaging itself. Moreover, the incorporation of additives can endow FGs with multifunctionality, making them ideal materials for packaging applications. Wang et al. embedded chitosan oligosaccharides into fish skin gelatin by electrospinning, followed by the addition of glucose for cross‐linking through the Maillard reaction.^[^ ^188^ ^]^ Then, an FGs packaging material with antimicrobial and antioxidant properties was developed. This material is characterized by water absorption and exhibits significant swelling behavior with a swelling rate of up to 954%. This advance highlights the potential of innovative bio‐based materials to improve the effectiveness and sustainability of food packaging. These FGs can extend the shelf life of fish by 2–4 days (**Figure** **13**a,b), demonstrating the potential application of FGs as packaging films for aquatic products. Water scarcity is a global issue, and the method of harvesting water from the air has emerged as one of the most promising solutions to alleviate water shortages. Qu et al. engineered a hydrogel fiber (HWTs) that integrates biomimetic principles from lizard skin and catfish epidermal mucus (Figure 13c).^[^ ^189^ ^]^ These FGs act as water transport channels, using their inherent directional pump to rapidly detach condensed water droplets from the condensing surface, thereby facilitating the collection of atmospheric water (Figure 13d). HWTs are networks formed by the interpenetration of sodium alginate and polyvinyl alcohol, featuring an arched structure with under‐crosslinked side chains covering the surface. The –OH and –COOH groups in the side chains of the FGs exhibited a strong affinity for water molecules. This affinity combined with the arched structure of the fiber provides a sufficient driving force to propel droplets from the condensation base to the FGs. In addition, the under‐crosslinked side chains of the FGs retained water between the molecular chains even after the droplets left the surface. This retention forms a precursor liquid film that acts as a lubricating layer, reducing the sliding resistance of subsequent droplets. This dual effect of directional pumping and friction‐reducing sliding facilitates sustainable water collection (Figure 13e). This design mimics natural systems and enhances the efficiency of water harvesting from the air, presenting a novel solution to water scarcity issues. Lin et al. achieved ordered alignment of PVA nanofibers using fluid‐induced techniques (Figure 13f) and collected FGs through a short‐distance coagulation bath technique (Figure 13g).^[^ ^190^ ^]^ Subsequently, the fibers were immersed in a salt solution for structural densification (Figure 13h,i). The resulting FGs exhibited ultra‐fast unidirectional water transport capabilities (Figure 13j), demonstrating their potential application in water purification.

## 4 Conclusions and Outlook

In this review, we discuss the latest strategies for enhancing the properties of FGs, including their mechanical performance, high conductivity, superior antibacterial and anti‐inflammatory capabilities, ECM‐like structure, and stimulus responsiveness. The mechanical properties of FGs are enhanced by the incorporation of exogenous fibers and orientation‐induced in situ fibrillation. Conductivity was improved by introducing electronically conductive fillers, creating ion‐conductive channels, and incorporating ionic gel fibers. The ECM‐like structure was achieved using four methods: cross‐linking of prefabricated fibers, interfacing of prefabricated fibers with prefabricated hydrogels, supramolecular assembly, and direct cross‐linking of natural fibers with polymers. Antibacterial and anti‐inflammatory properties are augmented by methods such as in situ gelation of drugs with prefabricated fibers, drug embedding within fibers, and in situ gelation of drugs with polymers. The intelligent response characteristics of FGs were developed by modulating pH, electromagnetism, and ultrasound. Optimized FGs have unique applications in tissue scaffolds, sensors, dressings, and water purification.

Despite significant advancements in the development of FGs in recent years, there are still some insurmountable challenges that hinder the seamless integration of FGs into practical application scenarios: a) *The Paradox Between Mechanical Properties and Failure Resistance*: modifying the composition or concentration of polymers to regulate the mechanical properties of FGs inevitably alters other characteristics. These modifications include changes in the functional groups within the hydrogel that influence cell adhesion density, alterations in the pore size of the internal fibers that impact nutrient transport, and an increase in ductility and flexibility resulting in reduced conductivity of the conductive FGs. Moreover, FGs with high mechanical properties degrade slowly, which poses challenges for their use in the biomedical field. Therefore, it is necessary to optimize the cross‐linking and fabrication strategies to enhance the mechanical properties without significantly reducing the functional efficacy.

b) *Complexity of Manufacturing Processes*: In general, the manufacturing process of high‐performance FGs is complex and difficult to scale for mass production. Furthermore, cumbersome production procedures and substantial solvent consumption do not correspond to green chemistry trends.

c) *Stability*: FGs are prone to dehydration during prolonged use, leading to functional failure. The introduction of humectants (such as glycerol and polyethylene glycol) and the application of elastomeric materials in encapsulation strategies can delay this process. However, most methods suffer from modulus mismatch and weak interfacial adhesion, which can easily result in delamination or rupture of the encapsulation layer. Therefore, the development of multifunctional FGs with enhanced water retention capabilities is crucial for overcoming these challenges.

*Development Trends*: First, microfluidic spinning allows precise control over the composition and structure of FGs. This method produces FGs biomaterials that emulate the multifunctionality and complexity of natural tissues. In addition, 3D bioprinting has demonstrated tremendous potential for enhancing the hierarchical complexity of FGs structures. Consequently, these two approaches have become the mainstream methods for fabricating FGs. Second, regarding functionality, FGs are evolving toward smart applications, transitioning from passive to active responses. By incorporating electrical, thermos‐sensitive, and magnetic properties, FGs have evolved into multifunctional integrated devices. For example, flexible sensors based on FGs can integrate multiple functions such as data processing, wireless transmission, and self‐powering, culminating in truly intelligent flexible electronic systems. The introduction of magnetically, electrically or optically responsive nano‐fillers to adjust the cellular microenvironment in response to external stimuli plays a crucial role in regulating cell growth and differentiation, as well as in directing tissue organization and function. This is crucial for the development of biomimetic scaffold. We anticipate that integrating multifunctional technologies and fostering interdisciplinary collaboration will lead to the development of innovative and functional FGs.

## Conflict of Interest

The authors declare no conflict of interest.
